# Association Between Rurality and Mortality: Observational Study of Spanish and United States Prehospital Emergency Care Cohorts

**DOI:** 10.3390/healthcare14070946

**Published:** 2026-04-04

**Authors:** Álvaro Astasio-Picado, José Luis Martín-Conty, Begoña Polonio-López, Cristina Rivera-Picón, Juan J. Bernal-Jiménez, Paula Álvarez Buitrago, Jorge García-Criado, María Cubillo-Jiménez, Juan F. Delgado Benito, Francisco Martín-Rodríguez, Ancor Sanz-García

**Affiliations:** 1 Intensive Care Unit, Hospital Virgen del Puerto, 10600 Plasencia, Spain; alvaro.astasio@uclm.es; 2 Faculty of Health Sciences, University of Castilla–La Mancha (UCLM), 45600 Talavera, Spainbegona.polonio@uclm.es (B.P.-L.); juanjose.bernal@uclm.es (J.J.B.-J.); 3 Technological Innovation Applied to Health Research Group (ITAS Group), Faculty of Health Sciences, University of de Castilla-La Mancha, 45600 Talavera de la Reina, Spain; 4 Evaluación de Cuidados de Salud (ECUSAL), Instituto de Investigación Sanitaria de Castilla-La Mancha (IDISCAM), 45004 Toledo, Spain; 5 Anaesthesia and Resuscitation Service, Complejo Hospitalario de Toledo, 45007 Toledo, Spain; palvarzb@sescam.jccm.es; 6 Department of Physiology and Pharmacology, Faculty of Medicine, University of Salamanca, 37007 Salamanca, Spain; jgarciacr@saludcastillayleon.es; 7 Castilla-Leon Health Service, Sanidad Castilla y Leon, University Hospital of Salamanca, 37007 Salamanca, Spain; mcubilloj@saludcastillayleon.es; 8 Advanced Life Support, Emergency Medical Services (SACYL), 47007 Valladolid, Spain; jdelgado@saludcastillayleon.es (J.F.D.B.); francisco.martin.rodriguez@uva.es (F.M.-R.); 9 Faculty of Medicine, University of Valladolid, 47011 Valladolid, Spain

**Keywords:** emergency medical services, rurality, urban, prehospital, mortality

## Abstract

**Background/Objectives:** Differences between rural and urban settings, as well as between emergency medical service (EMS) systems, may influence short-term mortality among patients attended in the prehospital setting. The aim of this study was to determine the associations of rurality and the US and Spanish EMS health systems with patient mortality. **Methods:** This was a multicenter, EMS-based, observational study involving a prospective dataset, the Salud de Castilla y Leon dataset (SACYL) from Spain, and a retrospective dataset, the National Emergency Medical Services Information System (NEMSIS) from the US. All consecutive EMS activations of adult patients (≥18 years) requiring high-priority transport to emergency departments were included in the analysis. The collected variables included demographic characteristics, EMS transport characteristics, case characteristics, and rural or urban origin. The primary outcome was 2-day, short-term mortality. **Results:** A total of 54,981 EMS activations were considered from both datasets. The mortality rate was 8.47% for rural areas and 11.8% for urban areas (*p* < 0.001). Multivariable analyses showed that mortality patterns differed according to geographic setting and EMS system. Male sex and the use of advanced life support were associated with higher odds of mortality in several models, while prehospital time intervals and call characteristics showed context- and system-dependent associations, including protective effects in specific subgroups. **Conclusions:** Short-term mortality differed between rural and urban settings, with heterogeneous patterns across EMS systems. These findings highlight the importance of considering both geographic context and system-level organizational characteristics when evaluating prehospital care and mortality outcomes.

## 1. Introduction

Prehospital care is a key pillar of healthcare systems, providing out-of-hospital Emergency Medical Services (EMSs). Its primary objective is to deliver timely and appropriate medical care in acute situations, including patient assessment, initial stabilization, and, when indicated, advanced life support interventions, before and during transport to a medical facility. This early management is critical in life-threatening conditions and can significantly influence patient outcomes. The care includes the initial assessment of the patient, the administration of first aid, and safe and rapid transportation to a hospital [1,2].

However, globally, EMS systems vary significantly depending on the available resources and the organizational structure. Differences in EMS strategic design and infrastructure between regions have been shown to affect response efficiency and equity, underlining the need to optimize services in both urban and rural settings to improve outcomes [3]. In the United States, the EMS operates in a decentralized manner, resulting in significant differences in organization, funding, and regulation between regions. This system includes various levels of care, ranging from basic life support (BLS) provided by basic responders to advanced life support (ALS) administered by paramedics trained to perform complex medical procedures [4,5]. The EMS is regulated by the National Highway Traffic Safety Administration (NHTSA), although each state is free to develop its own protocols, leading to substantial differences in the quality and scope of services [6,7,8].

In contrast, the EMS of Castilla y León, SACYL, Spain, features a more centralized structure. SACYL integrates a network of BLS and ALS units, with the involvement of physicians and registered emergency nurses (ERNs), who provide care both on scene and en route to the hospital. This centralized approach allows for greater cohesion in service delivery and a more uniform response to emergencies, regardless of location [9].

There are also significant disparities between rural and urban areas in terms of access to and quality of prehospital care services. In urban areas, higher population density and proximity to well-equipped medical centers facilitate faster response times [10,11]. However, in rural areas, resource shortages and longer distances can complicate timely and effective care. These factors contribute to notable differences in emergency medical care between rural and urban settings [12,13].

Thus, prehospital care is essential to healthcare systems worldwide, but its structure varies according to geographical and cultural context. Moreover, the comparison between the U.S. and SACYL highlights the diversity of approaches and the need to tailor emergency services to the characteristics of each population [14].

Therefore, there is a need for studies investigating how differences in the structure and functioning of EMS systems and patients’ living places impact clinical outcomes, particularly in terms of short-term mortality across different environments. The objective of this study was to determine the association of rurality with mortality in patients served by EMSs. Additionally, specific objectives were the following: (i) to explore the characteristics of rural patients by considering the association of variables with rurality for different healthcare systems (the U.S., the SACYL, and both); (ii) to explore the variables associated with mortality in rural patients in different healthcare systems (the U.S., the SACYL, and both); (iii) to explore this association differently in urban patients, again in different healthcare systems (the U.S., the SACYL, and both); and (iv) to explore the interaction between rurality and the health system.

## 2. Materials and Methods

### 2.1. Study Design

This was a multicenter, EMS-based, observational study involving a prospective dataset, the Salud de Castilla y Leon (SACYL) dataset, and a retrospective dataset, the National Emergency Medical Services Information System (NEMSIS) [14]. The simultaneous inclusion of two prehospital emergency care databases with distinct organizational and operational structures was intended to assess the generalizability of the association between rurality and mortality across different healthcare systems.

For the SACYL dataset, the study was approved by the local institutional research review board of the Public Health Service (reference: PI-049-19 and PI-GR-19-1258), and the research protocol was registered with the WHO International Clinical Trials Registry Platform (ISRCTN48326533 and ISRCTN49321933). For NEMSIS, the institutional research board granted a waiver/exemption owing to the use of deidentified data. We followed the guidelines of the Strengthening the Reporting of Observational Studies in Epidemiology (STROBE) Statement (Supplementary data p3).

### 2.2. Study Settings

The SACYL dataset was collected prospectively between 1 January 2018 and 31 December 2023 in four Spanish provinces (Burgos, Salamanca, Segovia, and Valladolid). The EMS is operated by the public health system and is integrated with the ALS (made up of 2 emergency medical technicians, an emergency registered nurse, and a physician), the Helicopter Emergency Medical Service (made up of an emergency registered nurse and a physician), and the BLS (made up of 2 emergency medical technicians). SACYL data were obtained from the public Emergency Medical Services system operated by the regional health authority (SACYL). Access to the dataset was granted following authorization by the corresponding institutional and ethics committees. The SACYL database is not publicly available, and its use is restricted to approved research projects in accordance with Spanish data protection regulations. The Spanish cohort comprises 11,713 EMS-attended cases collected prospectively in four provinces of the Castilla y León region. Together, these provinces cover a population of 995,137 inhabitants, resulting in an average of approximately 2000 EMS cases per year included in the study. These provinces are representative of a mixed rural–urban population. Valladolid and Burgos include medium-sized urban centers, while Salamanca and Segovia encompass a large proportion of rural and semi-rural areas with low population density. All Emergency Medical Services and receiving hospitals in the study area are fully integrated into the public health system and operate under the exclusive coordination of the regional Emergency Coordination Center (1-1-2). Consequently, the dataset captures all EMS activations within the defined geographic area, minimizing selection bias and enhancing population-level representativeness.

NEMSIS included retrospective data between 1 January 2018 and 31 December 2023, a United States of America dataset of EMS activations populated by more than twelve thousand EMS agencies throughout the United States. The EMS included in the dataset is integrated by ALS (made up of 2 paramedics), Helicopter Emergency Medical Service (made up of an emergency registered nurse and/or a physician), and BLS (made up of 2 emergency medical technicians). NEMSIS is a publicly available national registry, populated by more than 12,000 EMS agencies. De-identified NEMSIS data can be accessed by researchers upon request through the official NEMSIS website, in compliance with its data use agreement and federal privacy regulations.

### 2.3. Population

All consecutive adult EMS activations (≥18 years) evacuated with high priority to emergency departments were included in the analysis. Minors and all cases involving missing data were excluded.

### 2.4. Outcome

The primary outcome was short-term mortality. The SACYL was associated with 2-day mortality (all-cause and in- and out-of-hospital), and for the NEMSIS, short-term mortality was extrapolated from the ED and hospital disposition. The 2-day time frame was selected for two main reasons. First, early deaths are more likely to be directly associated with the clinical condition prompting Emergency Medical Services (EMS) activation and the prehospital care provided. Second, most early warning scores and risk stratification tools used in emergency and prehospital settings are designed to predict early clinical deterioration and short-term mortality rather than long-term outcomes. This outcome definition is consistent with previous studies in the prehospital and emergency medicine literature, which have demonstrated the relevance of early mortality as a clinically meaningful endpoint when evaluating EMS response and early risk prediction strategies [15,16]. Similar methodological approaches have been used in studies examining the relationship between prehospital care and early mortality, as well as the prognostic value of early warning scores and biomarkers in acute conditions [17,18]. Additionally, hospital admission and ICU admission were considered.

### 2.5. Variable Selection

Variables were selected considering their reliable harmonization across the two datasets to ensure methodological consistency and comparability between cohorts. The collected variables were grouped into three domains: (i) demographic variables, including age and sex, which are standard demographic factors consistently reported in both datasets and are well-established predictors of short-term mortality in emergency settings; (ii) EMS operational and transport characteristics, including the level of care provided (BLS or ALS), mission type (alert, on-scene support, or interfacility transfer), and time intervals (response time, on-scene time, transport time, and total EMS time)—these variables being selected due to their documented influence on patient outcomes and their relevance to evaluating system performance in prehospital emergency care; and (iii) case and contextual characteristics, including the chief complaint at the emergency call, suspected prehospital diagnosis, and geographic origin of the incident (urban vs. rural). Geographic classification was included to account for potential differences in EMS response times, access to advanced care, and population density. Because the two datasets use different geographic classification schemes, a harmonization process was applied. The SACYL database categorizes incident location exclusively as urban or rural. In contrast, the NEMSIS database includes four categories: urban, suburban, rural, and wilderness. To ensure comparability between datasets, suburban incidents in NEMSIS were grouped with urban areas, and wilderness incidents were grouped with rural areas, based on similarities in population density, infrastructure, and expected EMS response characteristics. This recoding approach has been used in prior cross-registry analyses and was chosen to minimize misclassification while preserving contextual relevance. The level of care reflects the expected clinical severity at dispatch. All cases included in the present analysis were patients who were evaluated on scene by an ALS team and subsequently transported to the emergency department, either by ALS or BLS units. Therefore, ALS involvement in this study indicates that the patient was considered to have sufficient potential severity to warrant advanced clinical evaluation.

### 2.6. Data Analysis

Descriptive results and the associations between the outcomes and the analyzed variables were assessed by a *t*-test, the Mann–Whitney U test, or the chi-square test, when appropriate. Absolute values and percentages were used for categorical variables, and median interquartile ranges (IQRs) were used for continuous variables because they did not follow a normal distribution. To determine the variables associated with mortality, a multivariate logistic regression was used. This process was also repeated but with a separate analysis of rural and urban patients. These results were represented via the multivariate logistic regression-derived odds ratios and 95% confidence intervals.

All calculations and analyses were performed by using our own codes, R packages, and base functions in R, version 4.2.2 (http://www.R-project.org accessed on 1 July 2025; the R Foundation for Statistical Computing, Vienna, Austria).

## 3. Results

**Figure 1:**
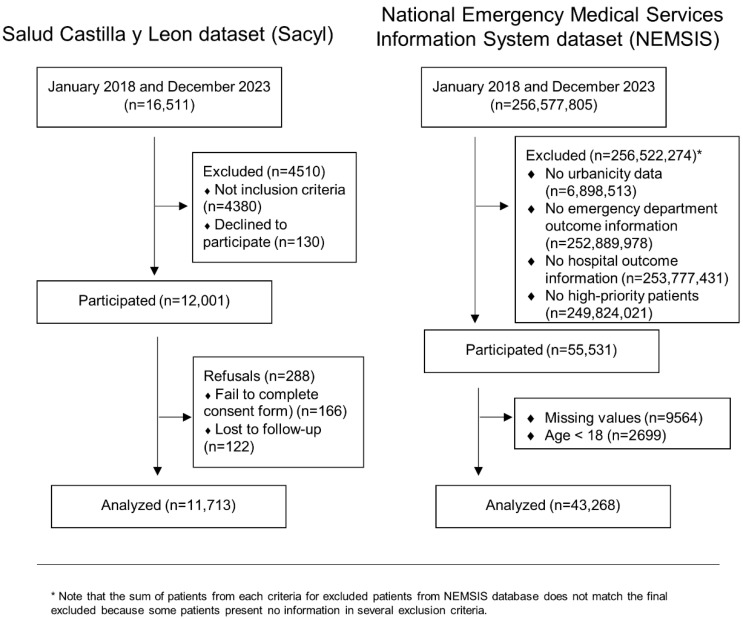
Flowchart.

A total of 11,713 EMS activations were considered from the SACYL dataset, and 43,268 were considered from the NEMSIS dataset (Figure 1). Descriptive tables of all cohorts combined (Table 1) and separately for the NEMSIS and SACYL (Supplementary Tables S1 and S2, respectively) revealed that the number of rural patients attended by the EMS was lower than the number of urban patients in both health systems (7069 patients (16.3%) for the NEMSIS and 2314 patients (19.8%) for the SACYL). Globally, the mortality rate according to living location was 795 (8.47%) for rural areas and 5358 (11.8%) for urban areas (*p* < 0.001). When considering mortality in each database, the NEMSIS presented a mortality rate of 528 (7.47%) for rural areas and 4196 (11.6%) for urban areas (*p* < 0.001). The SACYL, however, was not significantly different (*p* = 0.183). Age was significantly different between the rural and urban cohorts in both the databases and the global cohort; however, the global and NEMSIS results revealed that older rural patients were aged 62.00 and 61.7 years (for the global cohort and the NEMSIS cohort, respectively) vs. 61.4 and 60.4 years for the urban cohort (both *p* < 0.001). The SACYL database presented older urban patients: 65.4 vs. 62.8 for rural (*p* < 0.001). Regarding sex, no statistically significant differences were found for the global and NEMSIS databases. For the SACYL database, there were more males (1434, 62%) in rural areas than in urban areas (5403, 57.5%, *p* < 0.001). This difference between the NEMSIS and SACYL was also observed in terms of the level of care of the EMS units, specifically whether the unit was an ALS or a BLS. The NEMSIS presented a higher percentage of advanced life support for urban areas (33,015 (91.2%) vs. 5323 (75.3%) for rural areas), whereas for the SACYL, the opposite was observed: 1634 (70.6%) for rural areas and 5708 (60.7%) for urban areas (both *p* < 0.001). With respect to the complaints reported by dispatch, the NEMSIS presented differences between rural and urban areas for disease, social demand, and other causes, and the SACYL presented differences for labor accidents, social demand, and traffic. The rural or urban differences in the main organ or system affected showed that the NEMSIS database presented differences for cardiovascular, endocrine and metabolic, gastrointestinal, genitourinary, global/general, musculoskeletal/skin/trauma, neurologic, and pulmonary factors; the SACYL database presented differences only for musculoskeletal/skin/trauma and pulmonary factors. Finally, both cohorts presented greater numbers of alerts, transfers, and the totals for the rural patients than for urban patients (*p* < 0.001).

Comparisons between cohorts and groups resulting from the combination of cohorts and rural and urban areas can be found in Supplementary Tables S3 and S4.

### 3.1. Multivariate Logistic Regression for Mortality in the Rural Subset

**Figure 2:**
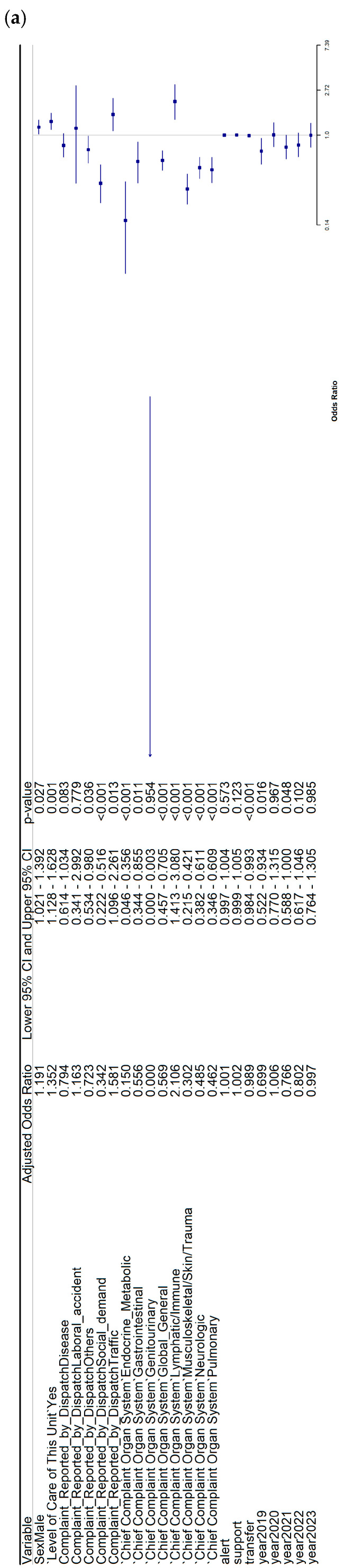

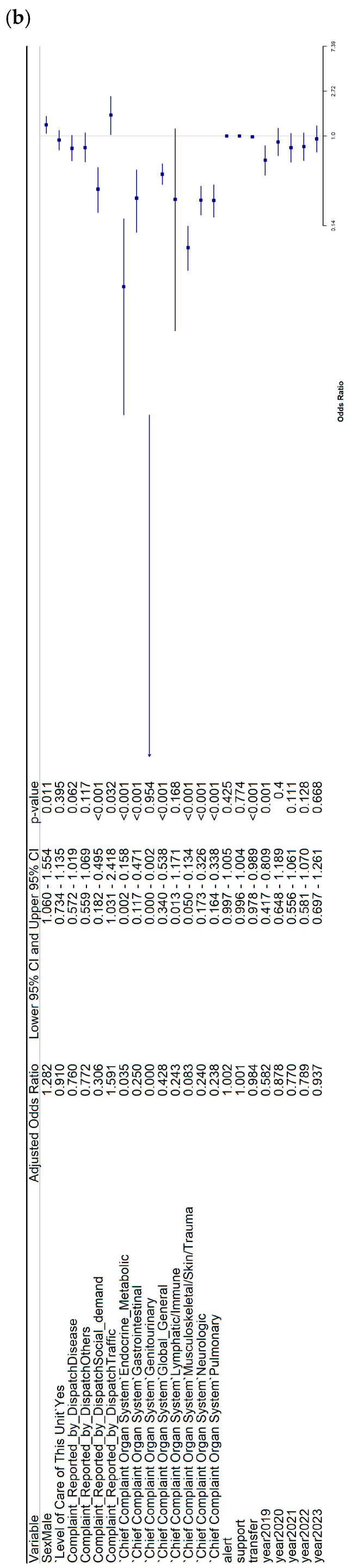

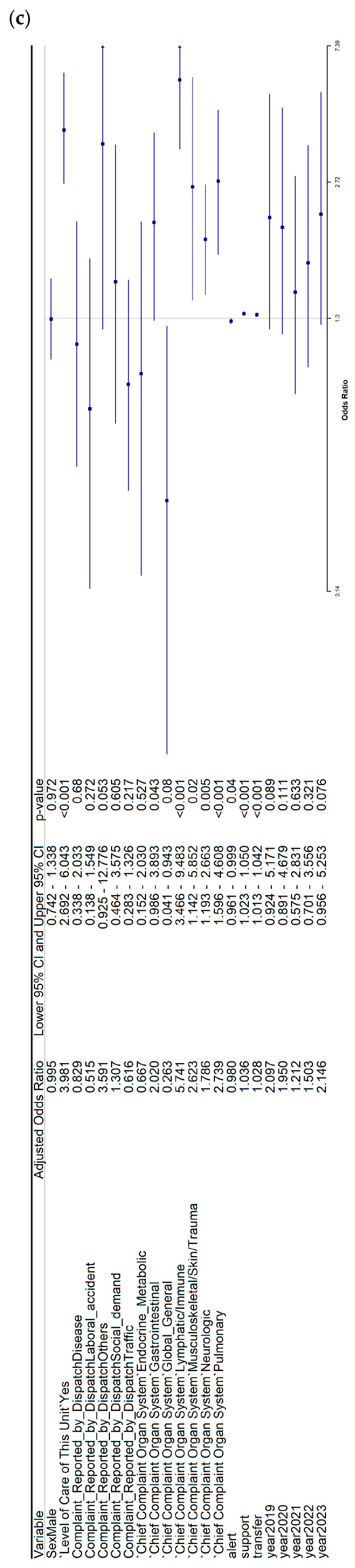
Adjusted odds ratios for mortality in rural patients. (**a**) Both cohorts, (**b**) NEMSIS, (**c**) SACYL.

The analyses of factors associated with mortality in rural patients are shown in Figure 2. In the pooled multivariate model including both cohorts (Figure 2a), male sex (OR: 1.19; 95% CI: 1.02–1.39) and the use of advanced life support (OR: 1.35; 95% CI: 1.13–1.63) were associated with higher odds of mortality, whereas longer transfer time (OR: 0.99; 95% CI: 0.98–0.99) and several complaint categories and organ-system groups were associated with lower odds of mortality.

In the NEMSIS rural cohort (Figure 2b), male sex (OR: 1.28; 95% CI: 1.06–1.55), traffic-related complaints (OR: 1.59; 95% CI: 1.03–2.42), and endocrine–metabolic conditions (OR: 1.54; 95% CI: 1.00–2.16) were associated with higher odds of mortality, while longer transfer time (OR: 0.98; 95% CI: 0.98–0.99), social demand, and several organ-system categories were associated with lower odds of mortality.

In the SACYL rural cohort (Figure 2c), the use of advanced life support (OR: 3.98; 95% CI: 2.69–6.04), longer support time (OR: 1.04; 95% CI: 1.02–1.05), longer transfer time (OR: 1.03; 95% CI: 1.01–1.04), and multiple organ-system categories were associated with higher odds of mortality, whereas longer total prehospital time was associated with lower odds of mortality (OR: 0.98; 95% CI: 0.96–0.99).

### 3.2. Multivariate Logistic Regression for Mortality in the Urban Subset

**Figure 3:**
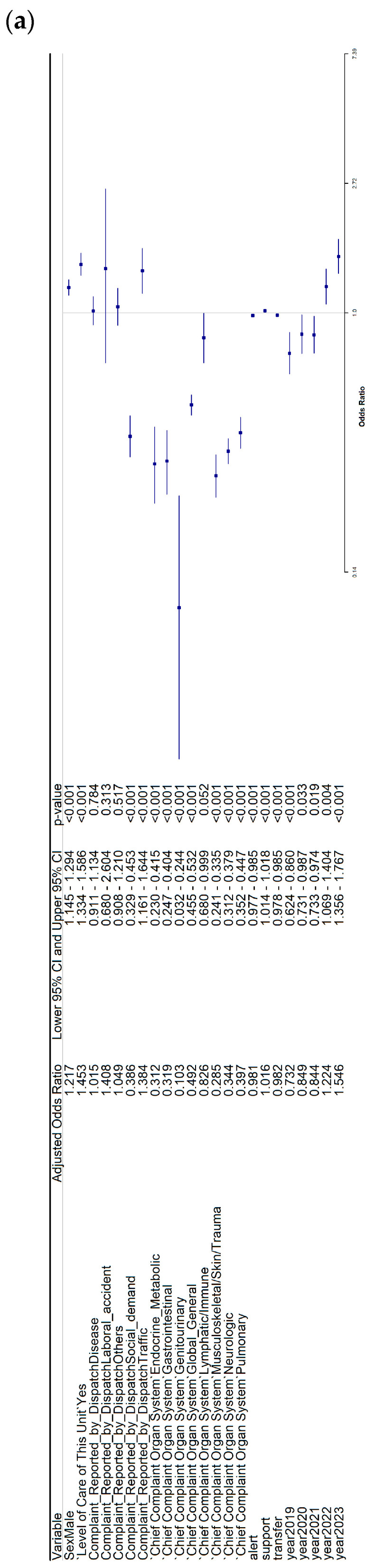

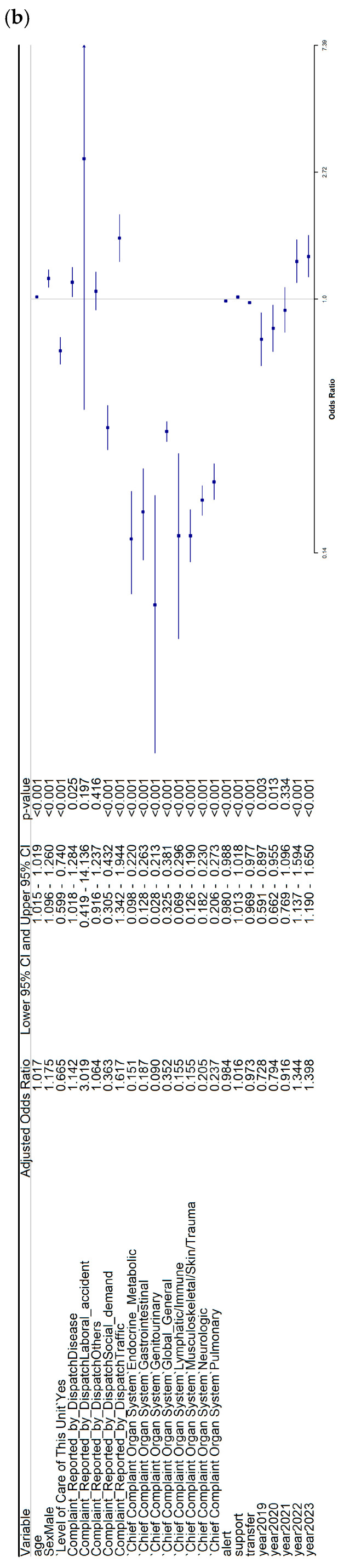

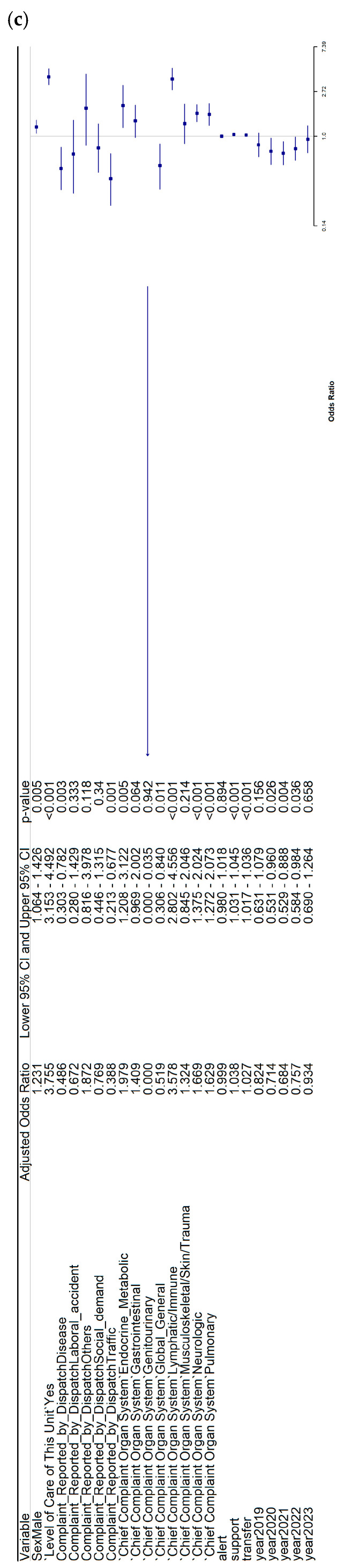
Adjusted odds ratios for mortality in urban patients. (**a**) Both cohorts, (**b**) NEMSIS, (**c**) SACYL.

The analyses of factors associated with mortality in urban patients are shown in Figure 3. In the pooled multivariate model including both cohorts (Figure 3a), male sex (OR: 1.22; 95% CI: 1.15–1.29), the use of advanced life support (OR: 1.45; 95% CI: 1.33–1.59), and longer support time (OR: 1.02; 95% CI: 1.01–1.02) were associated with higher odds of mortality. In addition, the years 2022 (OR: 1.22; 95% CI: 1.07–1.40) and 2023 (OR: 1.55; 95% CI: 1.36–1.77) were associated with higher odds of mortality.

Conversely, social demand (OR: 0.39; 95% CI: 0.33–0.45), traffic-related calls (OR: 0.38; 95% CI: 0.33–0.44), and multiple organ system categories, including endocrine–metabolic, gastrointestinal, genitourinary, global/general, musculoskeletal/skin/trauma, neurologic, and pulmonary, were associated with lower odds of mortality. Additionally, longer alert time (OR: 0.98; 95% CI: 0.98–0.99), longer transfer time (OR: 0.98; 95% CI: 0.98–0.99), and the years 2019, 2020, and 2021 were associated with lower odds of mortality.

In the urban NEMSIS cohort (Figure 3b), age (OR: 1.02; 95% CI: 1.01–1.02) and male sex (OR: 1.18; 95% CI: 1.10–1.27) were associated with higher odds of mortality. Moreover, disease-related calls (OR: 1.14; 95% CI: 1.02–1.28), traffic-related calls (OR: 1.62; 95% CI: 1.34–1.94), and the years 2022 (OR: 1.34; 95% CI: 1.14–1.59) and 2023 (OR: 1.40; 95% CI: 1.19–1.65) were associated with higher odds of mortality. By contrast, the use of advanced life support was associated with lower odds of mortality (OR: 0.67; 95% CI: 0.60–0.74), as were social demand and most organ system categories. In addition, longer alert time, longer transfer time, and the years 2019 and 2020 were associated with lower odds of mortality.

In the urban SACYL cohort (Figure 3c), male sex (OR: 1.23; 95% CI: 1.06–1.43), the use of advanced life support (OR: 3.76; 95% CI: 3.15–4.49), longer support time (OR: 1.04; 95% CI: 1.03–1.05), and longer transfer time (OR: 1.03; 95% CI: 1.02–1.04) were associated with higher odds of mortality. Regarding call reasons, disease-related calls (OR: 0.49; 95% CI: 0.33–0.78), social demand (OR: 0.77; 95% CI: 0.45–1.32), and traffic-related calls (OR: 0.39; 95% CI: 0.21–0.68) were associated with lower odds of mortality. With respect to organ system categories, endocrine–metabolic, global/general, lymphatic/immune, neurologic, and pulmonary conditions were associated with higher odds of mortality. Finally, the years 2021 (OR: 0.68; 95% CI: 0.53–0.89) and 2022 (OR: 0.76; 95% CI: 0.58–0.98) were associated with lower odds of mortality. The univariate analyses from which the previous results were derived can be found in Supplementary Tables S5–S10 for both cohorts of rural patients, NEMSIS rural patients, SACYL rural patients, both cohorts of urban patients, NEMSIS urban patients, and SACYL urban patients, respectively.

### 3.3. Multivariate Logistic Regression for Mortality Considering Both Rural and Urban Datasets

**Figure 4:**
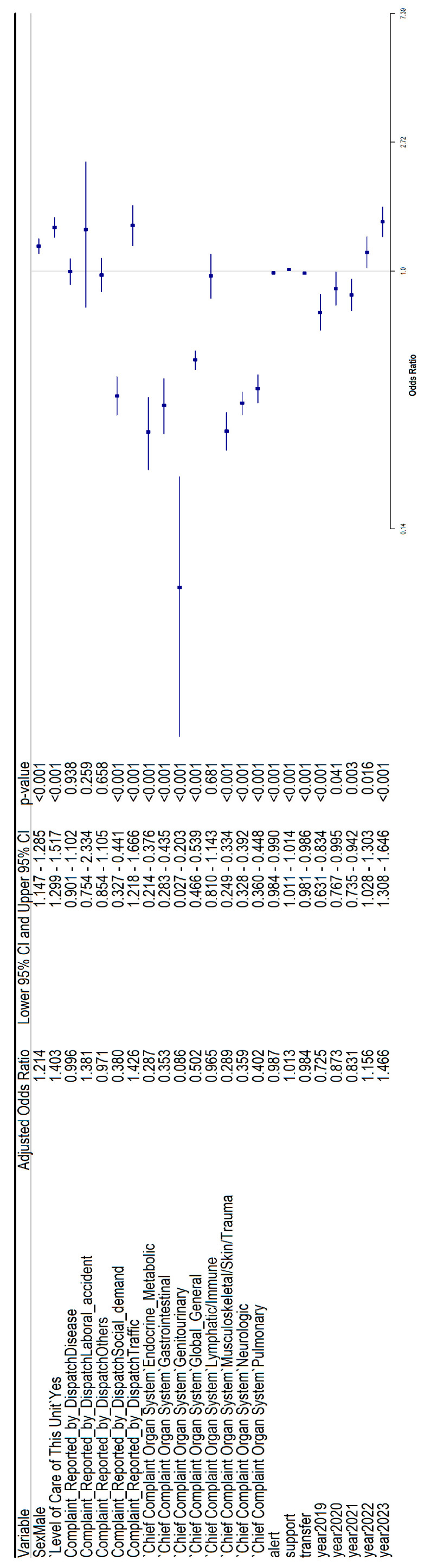
Adjusted odds ratios for both rural and urban datasets for mortality.

The previous analyses were also adjusted by using multivariate logistic regression for both datasets (rural and urban) together (Figure 4). The variables associated with mortality were sex, use of advanced life support, complaint reported by dispatch, chief complaint organ system, alert, transfer, total time, and year (2019 to 2023) (all *p* < 0.05).

### 3.4. Multivariate Logistic Regression Using a Four-Level Setting Variable

**Figure 5:**
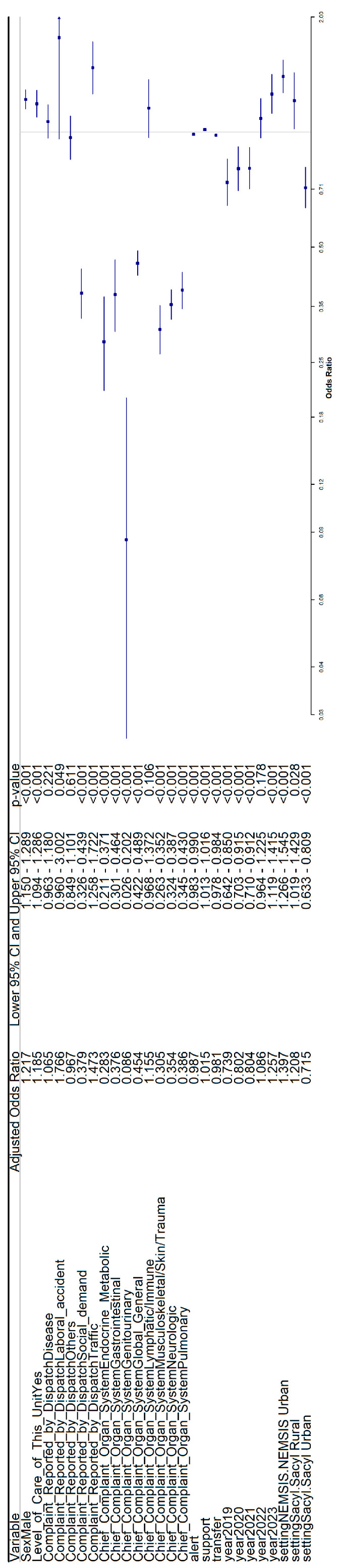
Adjusted odds ratios including the interaction term between rurality and the health system for mortality for both datasets.

In the logistic regression model using a combined four-level setting variable, with NEMSIS rural as the reference category (Figure 5), significant differences in the odds of the outcome were observed across groups. Compared with NEMSIS rural patients, those in NEMSIS urban areas had higher odds of the outcome (OR: 1.40, 95% CI: 1.27–1.54; *p* < 0.001). Similarly, patients in SACYL rural settings showed increased odds (OR: 1.21, 95% CI: 1.02–1.43; *p* = 0.028), but the same was not true for SACYL urban settings (OR: 0.71, 95% CI: 0.63–0.81; *p* < 0.001).

In inverse probability weighting (IPW) analyses performed for the NEMSIS cohort, the association between urban versus rural setting and mortality remained statistically significant and was greater in magnitude than in the conventional multivariable model. After weighting, urban patients had significantly higher odds of mortality compared with rural patients (OR: 3.07, 95% CI: 2.84–3.31; *p* < 0.001). In the SACYL cohort, IPW with Firth logistic regression was applied to account for potential confounding and extreme weights. Urban patients had significantly lower odds of mortality than rural patients (OR: 0.86, 95% CI: 0.81–0.91, *p* < 0.001), consistent with results from conventional multivariable logistic regression.

## 4. Discussion

In this multicenter observational study, short-term mortality outcomes in patients attended by EMS were analyzed using two large prehospital databases corresponding to healthcare systems with distinct organizational structures: SACYL (Spain) and NEMSIS (United States). The pooled analysis showed higher mortality in urban areas compared with rural areas, despite systematically longer operational times in rural settings. However, this overall pattern was not homogeneous across systems, highlighting the importance of considering organizational and care-related context when interpreting rural–urban differences in prehospital mortality.

When each database was analyzed separately, no statistically significant differences in mortality between rural and urban areas were observed in the SACYL system, whereas in NEMSIS, mortality was consistently higher in urban areas. These findings are consistent with previous studies evaluating EMS effectiveness in different contexts, which have reported that mortality may not differ between rural and urban settings after adjustment for structural and care-related factors, even when differences in response times and resources exist [19,20,21]. Beyond clinical outcomes, European evidence also points to rural–urban differences in the utilization of emergency services, including differential use of aeromedical transport, as well as community-level variations (e.g., first aid knowledge), which may influence prognosis before EMS arrival [22,23].

The centralized structure of the SACYL system may help explain the absence of mortality differences between rural and urban areas, as greater organizational and protocol homogeneity can mitigate care-related variability across territories [20,21]. In contrast, the NEMSIS analysis suggests that although transport times are longer in rural areas, mortality may be higher in urban areas due to service saturation and high population density [24,25,26].

From an operational perspective, rural patients experienced longer alert times, transport times, and total prehospital care times. This pattern is consistent with previous literature describing greater distances and geographic barriers in rural settings, which are associated with longer transport and total prehospital times [27]. However, in multivariable models, time intervals did not show a uniform association with increased mortality risk. In fact, some time variables (e.g., transport time) were associated with lower odds of mortality in specific subgroups, particularly in the pooled rural analysis and in NEMSIS. This finding suggests that “prehospital time” may act as a composite marker, reflecting not only distance but also operational decisions (resource allocation, on-scene stabilization, hospital destination selection) and care coordination. Therefore, its interpretation should be contextualized within the organizational structure of the system and the nature of the cases.

One of the most relevant findings of this study is the differential role of ALS in mortality. In SACYL, ALS was associated with higher odds of mortality in both rural and urban areas. Importantly, this association should be interpreted as a marker of greater baseline severity and clinical complexity rather than as a harmful effect of advanced prehospital care itself. These findings are consistent with previous studies documenting the negative impact of prolonged prehospital times on mortality [28,29]. In contrast, in the urban NEMSIS setting, ALS was associated with lower odds of mortality, which may reflect differences in activation criteria, clinical profiles attended by each level of care, and/or integration of ALS within the care continuum. These inter-system discrepancies reinforce the need to interpret ALS effects within the specific organizational framework of each EMS.

Male sex was consistently associated with higher mortality across several models in both rural and urban areas. Although causality cannot be established, this association may reflect differences in clinical profiles, baseline severity, or patterns of resource utilization and is consistent with previous literature highlighting the relevance of demographic characteristics in prognosis in emergency care settings [30,31].

Regarding call reasons, social-demand calls were consistently associated with lower odds of mortality, particularly in urban models, suggesting that these activations often correspond to low-acuity situations rather than potentially life-threatening conditions. Recent studies have also emphasized the role of contextual and organizational factors in shaping outcomes in emergency care across rural and urban environments [32,33]. In contrast, traffic-related calls showed a system- and context-dependent association with mortality: while they were associated with lower odds of mortality in the pooled urban model and in urban SACYL, they were associated with higher mortality risk in NEMSIS, both in rural and urban areas, likely reflecting greater injury severity and trauma burden in these scenarios. These findings underscore the need to interpret call reasons within the specific organizational and epidemiological framework of each EMS.

Analysis by affected organ or system revealed distinct patterns between NEMSIS and SACYL. In NEMSIS, mortality varied markedly according to the primary affected organ or system, potentially reflecting differences in access to specialized resources, care pathways, or disease burden depending on rural or urban context. In SACYL, although associations between organ system and mortality were less consistent across models, several categories were associated with higher mortality risk in both rural and urban areas, suggesting that the affected organ system remains a relevant prognostic determinant even within a more standardized organizational environment. Overall, these findings indicate that observed differences may be driven more by structural characteristics of the system than by rurality considered in isolation, highlighting the importance of interpreting the rural–urban dichotomy within the EMS framework [20,24,34].

Finally, the increase in mortality observed in 2022 and 2023 in NEMSIS may reflect indirect and persistent effects related to the COVID-19 pandemic, including delays in seeking care, accumulation of unmet healthcare needs, and sustained system pressure. Previous evidence suggests that unmet medical needs during the first wave of the pandemic were associated with adverse outcomes at later stages, which may help explain these temporal trends [33]. These findings highlight the complexity of factors influencing mortality in urban areas, where service saturation and population density may amplify the consequences of diagnostic and therapeutic delays. Overall, these data emphasize the need for a multidimensional approach when designing prehospital strategies, considering both risk and protective factors and adapting interventions to the specific needs of each population. Although causality cannot be established, these results underscore the importance of considering delayed and indirect pandemic-related effects when interpreting recent trends in prehospital mortality.

Among the main strengths of this study is its generalizability, derived from the use of two large cohorts from EMS systems with clearly distinct organizational structures and an extended study period. The comparison between SACYL and NEMSIS provides valuable insight into how organizational and contextual factors may influence outcomes beyond the simple rural–urban dichotomy.

Nevertheless, this study has limitations. First, the study period includes the COVID-19 pandemic years, which may have influenced EMS demand and performance. In addition, the variables included were limited to those that could be harmonized across databases, requiring simplification of categories (rurality, call reason, and organ system), which may affect comparative precision. Second, although the inclusion of two EMS systems is a strength, structural and operational differences between them require cautious interpretation of the findings and limit direct extrapolation to other healthcare contexts. Third, the absence of validated severity scores or comprehensive comorbidity data represents a potential source of residual confounding. However, by including level of care and chief complaint as proxies for acuity, we have attempted to mitigate this limitation to the extent possible, given the database constraints. Finally, due to the dual origin of the data, it was not possible to obtain relevant confounders such as comorbidities, healthcare access, or socioeconomic status, which are known to significantly affect outcomes. Future research should incorporate direct severity measures and more detailed clinical and socioeconomic data.

## 5. Conclusions

In conclusion, this study demonstrates that short-term mortality differs between rural and urban settings, with higher mortality observed in urban areas despite longer prehospital times in rural zones. Male sex, the use of advanced life support, and selected prehospital time intervals were identified as relevant factors associated with mortality, particularly in urban environments. These findings highlight the relevance of geographic context when evaluating prehospital outcomes while also suggesting that system-level organizational characteristics may modulate the magnitude and direction of rural–urban differences. Overall, the results underscore the importance of adapting prehospital care strategies to the specific demands and organizational structures of rural and urban EMS systems in order to optimize emergency care delivery and improve patient outcomes.

## Figures and Tables

**Table 1:** Patient characteristics according to living place (rural or urban).

	Rural	Urban	Odds Ratio [95%CI]	*p*-Value
	N = 9383	N = 45,598		
Age	62.0 (19.6)	61.4 (19.6)	1.00 [1.00; 1.00]	0.010
Sex:				
Female	3889 (41.4%)	19,210 (42.1%)	Ref.	Ref.
Male	5494 (58.6%)	26,388 (57.9%)	0.97 [0.93; 1.02]	0.223
Level of care of the EMS unit:				
Basic life support	2426 (25.9%)	6875 (15.1%)	Ref.	Ref.
Advanced life support	6957 (74.1%)	38,723 (84.9%)	1.96 [1.86; 2.07]	<0.001
Complaint reported by dispatch:				
Causal accident	1374 (14.6%)	6066 (13.3%)	Ref.	Ref.
Disease	4824 (51.4%)	27,186 (59.6%)	1.28 [1.19; 1.36]	<0.001
Laboral accident	62 (0.66%)	138 (0.30%)	0.50 [0.37; 0.69]	<0.001
Others	1607 (17.1%)	4249 (9.32%)	0.60 [0.55; 0.65]	<0.001
Social demand	896 (9.55%)	5585 (12.2%)	1.41 [1.29; 1.55]	<0.001
Traffic	620 (6.61%)	2374 (5.21%)	0.87 [0.78; 0.96]	0.009
Chief complaint organ system:				
Cardiovascular	2484 (26.5%)	11,824 (25.9%)	Ref.	Ref.
Endocrine metabolic	209 (2.23%)	773 (1.70%)	0.78 [0.66; 0.91]	0.002
Gastrointestinal	338 (3.60%)	1189 (2.61%)	0.74 [0.65; 0.84]	<0.001
Genitourinary	92 (0.98%)	281 (0.62%)	0.64 [0.51; 0.82]	<0.001
Global general	2078 (22.1%)	14,182 (31.1%)	1.43 [1.35; 1.53]	<0.001
Lymphatic/immune	165 (1.76%)	823 (1.80%)	1.05 [0.88; 1.25]	0.610
Musculoskeletal/skin/trauma	1415 (15.1%)	3967 (8.70%)	0.59 [0.55; 0.63]	<0.001
Neurologic	1595 (17.0%)	8513 (18.7%)	1.12 [1.05; 1.20]	0.001
Pulmonary	1007 (10.7%)	4046 (8.87%)	0.84 [0.78; 0.92]	<0.001
Hospital admission:				
No	6943 (74.0%)	20,475 (44.9%)	Ref.	Ref.
Yes	2440 (26.0%)	25,123 (55.1%)	3.49 [3.32; 3.67]	<0.001
ICU admission:				
No	8073 (86.0%)	41,628 (91.3%)	Ref.	Ref.
Yes	1310 (14.0%)	3970 (8.71%)	0.59 [0.55; 0.63]	<0.001
Hospital mortality:				
No	8612 (91.8%)	40,525 (88.9%)	Ref.	Ref.
Yes	771 (8.22%)	5073 (11.1%)	1.40 [1.29; 1.51]	<0.001
Total mortality:				
No	8588 (91.5%)	40,240 (88.2%)	Ref.	Ref.
Yes	795 (8.47%)	5358 (11.8%)	1.44 [1.33; 1.56]	<0.001
Alert time	18.0 (20.9)	12.4 (25.4)	0.99 [0.99; 0.99]	<0.001
Support time	24.2 (20.6)	23.9 (16.7)	1.00 [1.00; 1.00]	0.065
Transfer time	24.6 (27.4)	15.6 (16.5)	0.98 [0.98; 0.98]	<0.001
Total time	66.9 (48.9)	51.8 (42.0)	0.99 [0.99; 0.99]	<0.001
Year:				
2018	1198 (12.8%)	2697 (5.91%)	Ref.	Ref.
2019	1392 (14.8%)	4627 (10.1%)	1.48 [1.35; 1.62]	<0.001
2020	1490 (15.9%)	6409 (14.1%)	1.91 [1.75; 2.09]	<0.001
2021	1884 (20.1%)	8710 (19.1%)	2.05 [1.89; 2.23]	<0.001
2022	1888 (20.1%)	9997 (21.9%)	2.35 [2.16; 2.56]	<0.001
2023	1531 (16.3%)	13,158 (28.9%)	3.82 [3.50; 4.16]	<0.001
Cohort:				
NEMSIS	7069 (75.3%)	36,199 (79.4%)	Ref.	Ref.
SACYL	2314 (24.7%)	9399 (20.6%)	0.79 [0.75; 0.84]	<0.001

## Data Availability

The data used in this study are not publicly available due to privacy and ethical restrictions. De-identified data from the SACYL and NEMSIS datasets may be made available from the corresponding author upon reasonable request and subject to approval by the relevant data custodians and ethics committees.

## Supplementary Materials

The following supporting information can be downloaded at https://www.mdpi.com/article/10.3390/healthcare14070946/s1. Table S1. Patients’ characteristics according to living place (rural or urban) for the NEMSIS database. Table S2. Patients’ characteristics according to living place (rural or urban) for the SACYL database. Table S3. Patients’ characteristics according to cohort (NEMSIS vs. SACYL). Table S4. Comparison between the groups resulting from the combination of cohorts and rural and urban areas. Table S5. Patients’ characteristics according to mortality for both cohorts of rural patients. Table S6. Patients’ characteristics according to mortality for NEMSIS rural patients. Table S7. Patients’ characteristics according to mortality for SACYL rural patients. Table S8. Patients’ characteristics according to mortality for both cohorts of urban patients. Table S9. Patients’ characteristics according to mortality for NEMSIS urban patients. Table S10. Patients’ characteristics according to mortality for SACYL urban patients [35].

## Abbreviations

The following abbreviations are used in this manuscript:

ALS	Advanced Life Support
BLS	Basic Life Support
CI	Confidence Interval
COVID-19	Coronavirus Disease 2019
ED	Emergency Department
EMS	Emergency Medical Services
ERN	Emergency Registered Nurse
ICU	Intensive Care Unit
IQR	Interquartile Range
ISRCTN	International Standard Randomized Controlled Trial Number
NEMSIS	National Emergency Medical Services Information System
NHTSA	National Highway Traffic Safety Administration
OR	Odds Ratio
PI	Project Identifier (Ethics Approval Code)
R	Statistical Software R (version 4.2.2)
SACYL	Salud de Castilla y León
STROBE	Strengthening the Reporting of Observational Studies in Epidemiology
USA	United States of America
WHO	World Health Organization
