# Supplementary material for: Association Between Rurality and Mortality: Observational Study of Spanish and United States Prehospital Emergency Care Cohorts
Source: Healthcare (Basel). 2026 Apr 4;14(7):946. doi: 10.3390/healthcare14070946 (PMC13073158; doi:10.3390/healthcare14070946)
Supplement: Supplementary file 1 [file healthcare-14-00946-s001.zip › healthcare-4181411-supplementary.pdf]

Supplementary data

Table of contents

Supplementary methods…………………………………………………………………………………3
Strengthening the Reporting of Observational Studies in Epidemiology (STROBE) Statement……………………..…………………………………3
Data collection, Missing values, sample size calculation, software…..………..5
Supplementary Results…..………………………………………………………..6
- Supplementary table S1. Patients' characteristics according to living place (rural or urban) for NEMSIS database………..…………………….……………………………………..6
- Supplementary table S2. Patients' characteristics according to living place (rural or urban) for Sacyl database………..…………………….……………………………………..8
- Supplementary table S3. Patients' characteristics according to cohort (NEMSIS vs Sacyl)…………………………………………………………………..10
- Supplementary table S4. Comparison between the groups resulting from the combination of cohorts and rural and urban…………………………………...12
- Supplementary table S5. Patients' characteristics according to mortality for both cohorts rural patients……………………………………………………14
- Supplementary table S6. Patients' characteristics according to mortality for NEMSIS rural patients…………………………………………………………16
- Supplementary table S7. Patients' characteristics according to mortality for Sacyl rural patients..………………………………………………………18
- Supplementary table S8. Patients' characteristics according to mortality for both cohorts urban patients..…………………………………………………20
- Supplementary table S9. Patients' characteristics according to mortality for NEMSIS urban patients..………………………………………………………22
- Supplementary table S10. Patients' characteristics according to mortality for Sacyl urban patients.…………………………………………………………24
References..26

Supplementary Methods

STROBE[1] Statement—checklist of items that should be included in reports of observational studies

	Item No	Recommendation	Page No
**Title and abstract**	1	(*a*) Indicate the study's design with a commonly used term in the title or the abstract	1
		(*b*) Provide in the abstract an informative and balanced summary of what was done and what was found	3
**Introduction**			
Background/rationale	2	Explain the scientific background and rationale for the investigation being reported	4
Objectives	3	State specific objectives, including any prespecified hypotheses	4
**Methods**			
Study design	4	Present key elements of study design early in the paper	6
Setting	5	Describe the setting, locations, and relevant dates, including periods of recruitment, exposure, follow-up, and data collection	6
Participants	6	(*a*) *Cohort study*—Give the eligibility criteria, and the sources and methods of selection of participants. Describe methods of follow-up<br>*Case-control study*—Give the eligibility criteria, and the sources and methods of case ascertainment and control selection. Give the rationale for the choice of cases and controls<br>*Cross-sectional study*—Give the eligibility criteria, and the sources and methods of selection of participants	6
		(*b*) *Cohort study*—For matched studies, give matching criteria and number of exposed and unexposed<br>*Case-control study*—For matched studies, give matching criteria and the number of controls per case	6
Variables	7	Clearly define all outcomes, exposures, predictors, potential confounders, and effect modifiers. Give diagnostic criteria, if applicable	6
Data sources/ measurement	8*	For each variable of interest, give sources of data and details of methods of assessment (measurement). Describe comparability of assessment methods if there is more than one group	*6*
Bias	9	Describe any efforts to address potential sources of bias	7
Study size	10	Explain how the study size was arrived at	7
Quantitative variables	11	Explain how quantitative variables were handled in the analyses. If applicable, describe which groupings were chosen and why	7
Statistical methods	12	(*a*) Describe all statistical methods, including those used to control for confounding	7
		(*b*) Describe any methods used to examine subgroups and interactions	7
		(*c*) Explain how missing data were addressed	7
		(*d*) *Cohort study*—If applicable, explain how loss to follow-up was addressed<br>*Case-control study*—If applicable, explain how matching of cases and controls was addressed<br>*Cross-sectional study*—If applicable, describe analytical methods taking account of sampling strategy	7
		(*e*) Describe any sensitivity analyses	7

Continued on next page

Results

Participants	13*	(a) Report numbers of individuals at each stage of study—eg numbers potentially eligible, examined for eligibility, confirmed eligible, included in the study, completing follow-up, and analysed	9
		(b) Give reasons for non-participation at each stage	9
		(c) Consider use of a flow diagram	9
Descriptive data	14*	(a) Give characteristics of study participants (eg demographic, clinical, social) and information on exposures and potential confounders	9
		(b) Indicate number of participants with missing data for each variable of interest	9
		(c) *Cohort study*—Summarise follow-up time (eg, average and total amount)	9
Outcome data	15*	*Cohort study*—Report numbers of outcome events or summary measures over time	*9*
		Case-control study—Report numbers in each exposure category, or summary measures of exposure	*9*
		Cross-sectional study—Report numbers of outcome events or summary measures	*9*
Main results	16	(*a*) Give unadjusted estimates and, if applicable, confounder-adjusted estimates and their precision (eg, 95% confidence interval). Make clear which confounders were adjusted for and why they were included	9
		(*b*) Report category boundaries when continuous variables were categorized	9
		(*c*) If relevant, consider translating estimates of relative risk into absolute risk for a meaningful time period	9
Other analyses	17	Report other analyses done—eg analyses of subgroups and interactions, and sensitivity analyses	9
**Discussion**			
Key results	18	Summarise key results with reference to study objectives	11-12
Limitations	19	Discuss limitations of the study, taking into account sources of potential bias or imprecision. Discuss both direction and magnitude of any potential bias	12-13
Interpretation	20	Give a cautious overall interpretation of results considering objectives, limitations, multiplicity of analyses, results from similar studies, and other relevant evidence	11,12,13
Generalisability	21	Discuss the generalisability (external validity) of the study results	11,12,13
**Other information**			
Funding	22	Give the source of funding and the role of the funders for the present study and, if applicable, for the original study on which the present article is based	2

*Give information separately for cases and controls in case-control studies and, if applicable, for exposed and unexposed groups in cohort and cross-sectional studies.

Note: An Explanation and Elaboration article discusses each checklist item and gives methodological background and published examples of transparent reporting. The STROBE checklist is best used in conjunction with this article (freely available on the Web sites of PLoS Medicine at http://www.plosmedicine.org/, Annals of Internal Medicine at http://www.annals.org/, and Epidemiology at http://www.epidem.com/). Information on the STROBE Initiative is available at www.strobe-statement.org.

Data collection and missing values
Sacyl data was collected and registered in a database generated with the IBM SPSS Statistics for Apple version 20.0 software. (IBM Corp, Armonk USA). The caseload entry system was test-run to delete unclear or ambiguous items and to verify the adequacy of the data gathering system. Missing values were completely at random, therefore a listwise deletion method was used since it does not induce to biased means, variances or regression weights modification.
NEMSIS Data was collected from NEMSIS (https://nemsis.org/). The data present missing values but as occurred in the Sacyl database, missing values were completely at random.

Sample Size
The sample size needed for the present study is n=185 based on the following considerations: a statistical power (1 - β) of 80%, significant level (α) of p = 0.05, a proportion of sample in case group (q1) = 0.1, and an estimated Odds ratio of 2.

Software
All calculations and analyses were performed by using our own codes, R packages and base functions in R, version 4.2.2 (http://www.R-project.org; the R Foundation for Statistical Computing, Vienna, Austria).

Table S1. Patients' characteristics according to living place (rural or urban) for NEMSIS database

	**Rural** *N=7069*	**Urban** *N=36199*	**Odds ratio [95%CI]**	**pvalue**
age	61.7 (19.7)	60.4 (19.6)	1.00 [1.00;1.00]	<0.001
Sex:				
Female	3009 (42.6%)	15214 (42.0%)	Ref.	Ref.
Male	4060 (57.4%)	20985 (58.0%)	1.02 [0.97;1.08]	0.403
Level of Care of The EMS Unit:				
Basic life support	1746 (24.7%)	3184 (8.80%)	Ref.	Ref.
Advanced life support	5323 (75.3%)	33015 (91.2%)	3.40 [3.19;3.63]	<0.001
Complaint_Reported_by_Dispatch:				
causal_accident	1256 (17.8%)	5642 (15.6%)	Ref.	Ref.
Disease	3067 (43.4%)	19469 (53.8%)	1.41 [1.31;1.52]	<0.001
Laboral_accident	3 (0.04%)	9 (0.02%)	0.65 [0.19;3.06]	0.538
Others	1586 (22.4%)	4183 (11.6%)	0.59 [0.54;0.64]	<0.001
Social_demand	759 (10.7%)	4925 (13.6%)	1.44 [1.31;1.59]	<0.001
Traffic	398 (5.63%)	1971 (5.44%)	1.10 [0.97;1.25]	0.122
Chief Complaint Organ System:				
Cardiovascular	1605 (22.7%)	8063 (22.3%)	Ref.	Ref.
Endocrine_Metabolic	164 (2.32%)	607 (1.68%)	0.74 [0.62;0.88]	0.001
Gastrointestinal	240 (3.40%)	766 (2.12%)	0.64 [0.54;0.74]	<0.001
Genitourinary	88 (1.24%)	253 (0.70%)	0.57 [0.45;0.74]	<0.001
Global_General	1903 (26.9%)	13422 (37.1%)	1.40 [1.31;1.51]	<0.001
Lymphatic/Immune	30 (0.42%)	199 (0.55%)	1.31 [0.91;1.98]	0.154
Musculoskeletal/Skin/Trauma	982 (13.9%)	2887 (7.98%)	0.59 [0.53;0.64]	<0.001
Neurologic	1179 (16.7%)	6691 (18.5%)	1.13 [1.04;1.23]	0.003
Pulmonary	878 (12.4%)	3311 (9.15%)	0.75 [0.69;0.82]	<0.001
Hospital:				
No	6008 (85.0%)	16052 (44.3%)	Ref.	Ref.
Yes	1061 (15.0%)	20147 (55.7%)	7.11 [6.64;7.61]	<0.001
ICU:				

	Rural<br>*N=7069*	Urban<br>*N=36199*	Odds ratio [95%CI]	pvalue
No	6453 (91.3%)	34201 (94.5%)	Ref.	Ref.
Yes	616 (8.71%)	1998 (5.52%)	0.61 [0.56;0.67]	<0.001
hospital_mortality:				
No	6541 (92.5%)	32003 (88.4%)	Ref.	Ref.
Yes	528 (7.47%)	4196 (11.6%)	1.62 [1.48;1.79]	<0.001
total_mortality:				
No	6532 (92.4%)	31800 (87.8%)	Ref.	Ref.
Yes	537 (7.60%)	4399 (12.2%)	1.68 [1.53;1.85]	<0.001
alert	16.5 (23.4)	13.1 (28.4)	1.00 [1.00;1.00]	<0.001
support	22.8 (22.7)	22.4 (17.8)	1.00 [1.00;1.00]	0.135
transfer	25.8 (30.9)	16.9 (18.0)	0.98 [0.98;0.98]	<0.001
total	65.0 (55.1)	52.4 (46.7)	1.00 [1.00;1.00]	<0.001
year:				
2018	1090 (15.4%)	1797 (4.96%)	Ref.	Ref.
2019	1167 (16.5%)	2779 (7.68%)	1.44 [1.30;1.60]	<0.001
2020	1182 (16.7%)	5412 (15.0%)	2.78 [2.52;3.06]	<0.001
2021	1046 (14.8%)	6218 (17.2%)	3.61 [3.26;3.98]	<0.001
2022	1307 (18.5%)	7720 (21.3%)	3.58 [3.26;3.94]	<0.001
2023	1277 (18.1%)	12273 (33.9%)	5.83 [5.30;6.41]	<0.001

Table S2. Patients' characteristics according to living place (rural or urban) for Sacyl database

	**Rural** *N=2314*	**Urban** *N=9399*	**Odds ratio [95%CI]**	**pvalue**
age	62.8 (19.5)	65.4 (19.1)	1.01 [1.00;1.01]	<0.001
Sex:				
Female	880 (38.0%)	3996 (42.5%)	Ref.	Ref.
Male	1434 (62.0%)	5403 (57.5%)	0.83 [0.76;0.91]	<0.001
Level of Care of The EMS Unit:				
Basic life support	680 (29.4%)	3691 (39.3%)	Ref.	Ref.
Advanced life support	1634 (70.6%)	5708 (60.7%)	0.64 [0.58;0.71]	<0.001
Complaint_Reported_by_Dispatch:				
causal_accident	118 (5.10%)	424 (4.51%)	Ref.	Ref.
Disease	1757 (75.9%)	7717 (82.1%)	1.22 [0.99;1.51]	0.065
Laboral_accident	59 (2.55%)	129 (1.37%)	0.61 [0.42;0.88]	0.009
Others	21 (0.91%)	66 (0.70%)	0.87 [0.52;1.52]	0.615
Social_demand	137 (5.92%)	660 (7.02%)	1.34 [1.02;1.76]	0.037
Traffic	222 (9.59%)	403 (4.29%)	0.51 [0.39;0.66]	<0.001
Chief Complaint Organ System:				
Cardiovascular	879 (38.0%)	3761 (40.0%)	Ref.	Ref.
Endocrine_Metabolic	45 (1.94%)	166 (1.77%)	0.86 [0.62;1.22]	0.388
Gastrointestinal	98 (4.24%)	423 (4.50%)	1.01 [0.80;1.28]	0.949
Genitourinary	4 (0.17%)	28 (0.30%)	1.58 [0.62;5.48]	0.369
Global_General	175 (7.56%)	760 (8.09%)	1.01 [0.85;1.22]	0.876
Lymphatic/Immune	135 (5.83%)	624 (6.64%)	1.08 [0.89;1.32]	0.452
Musculoskeletal/Skin/Trauma	433 (18.7%)	1080 (11.5%)	0.58 [0.51;0.67]	<0.001
Neurologic	416 (18.0%)	1822 (19.4%)	1.02 [0.90;1.17]	0.725
Pulmonary	129 (5.57%)	735 (7.82%)	1.33 [1.09;1.63]	0.004
Hospital:				
No	935 (40.4%)	4423 (47.1%)	Ref.	Ref.
Yes	1379 (59.6%)	4976 (52.9%)	0.76 [0.70;0.84]	<0.001
ICU:				
No	1620 (70.0%)	7427 (79.0%)	Ref.	Ref.
Yes	694 (30.0%)	1972 (21.0%)	0.62 [0.56;0.69]	<0.001
hospital_mortality:				
No	2071 (89.5%)	8522 (90.7%)	Ref.	Ref.
Yes	243 (10.5%)	877 (9.33%)	0.88 [0.76;1.02]	0.089
total_mortality:				
No	2056 (88.9%)	8440 (89.8%)	Ref.	Ref.
Yes	258 (11.1%)	959 (10.2%)	0.91 [0.78;1.05]	0.183
alert	22.8 (8.68)	9.61 (3.64)	0.64 [0.63;0.65]	<0.001
support	28.7 (11.1)	29.5 (10.1)	1.01 [1.00;1.01]	0.001
transfer	21.1 (11.1)	10.4 (6.65)	0.87 [0.86;0.87]	<0.001
total	72.7 (19.5)	49.5 (12.9)	0.91 [0.91;0.92]	<0.001

	**Rural** *N=2314*	**Urban** *N=9399*	**Odds ratio [95%CI]**	**pvalue**
year:				
2018	108 (4.67%)	900 (9.58%)	Ref.	Ref.
2019	225 (9.72%)	1848 (19.7%)	0.99 [0.77;1.25]	0.911
2020	308 (13.3%)	997 (10.6%)	0.39 [0.31;0.49]	<0.001
2021	838 (36.2%)	2492 (26.5%)	0.36 [0.29;0.44]	<0.001
2022	581 (25.1%)	2277 (24.2%)	0.47 [0.38;0.58]	<0.001
2023	254 (11.0%)	885 (9.42%)	0.42 [0.33;0.53]	<0.001
cohort: Sacyl	2314 (100%)	9399 (100%)	Ref.	Ref.

Table S3. Patients' characteristics according to cohort (NEMSIS vs Sacyl)

	NEMSIS N=43268	Sacyl N=11713	Odds ratio [95%CI]	pvalue
age	60.6 (19.6)	64.9 (19.2)	1.01 [1.01;1.01]	<0.001
Sex:				
Female	18223 (42.1%)	4876 (41.6%)	Ref.	Ref.
Male	25045 (57.9%)	6837 (58.4%)	1.02 [0.98;1.06]	0.343
Level of Care of The EMS Unit:				
Basic life support	4930 (11.4%)	4371 (37.3%)	Ref.	Ref.
Advanced life support	38338 (88.6%)	7342 (62.7%)	0.22 [0.21;0.23]	<0.001
Zone:				
Rural	7069 (16.3%)	2314 (19.8%)	Ref.	Ref.
Urban	36199 (83.7%)	9399 (80.2%)	0.79 [0.75;0.84]	<0.001
Complaint_Reported_by_Dispatch:				
causal_accident	6898 (15.9%)	542 (4.63%)	Ref.	Ref.
Disease	22536 (52.1%)	9474 (80.9%)	1.41 [1.31;1.52]	<0.001
Laboral_accident	12 (0.03%)	188 (1.61%)	197 [114;375]	<0.001
Others	5769 (13.3%)	87 (0.74%)	0.19 [0.15;0.24]	<0.001
Social_demand	5684 (13.1%)	797 (6.80%)	1.44 [1.31;1.59]	<0.001
Traffic	2369 (5.48%)	625 (5.34%)	3.36 [2.97;3.80]	<0.001
Chief Complaint Organ System:				
Cardiovascular	9668 (22.3%)	4640 (39.6%)	Ref.	Ref.
Endocrine_Metabolic	771 (1.78%)	211 (1.80%)	0.57 [0.49;0.67]	<0.001
Gastrointestinal	1006 (2.33%)	521 (4.45%)	1.08 [0.96;1.21]	0.181
Genitourinary	341 (0.79%)	32 (0.27%)	0.20 [0.13;0.28]	<0.001
Global_General	15325 (35.4%)	935 (7.98%)	0.13 [0.12;0.14]	<0.001
Lymphatic/Immune	229 (0.53%)	759 (6.48%)	6.90 [5.94;8.05]	<0.001
Musculoskeletal/Skin/Trauma	3869 (8.94%)	1513 (12.9%)	0.81 [0.76;0.87]	<0.001
Neurologic	7870 (18.2%)	2238 (19.1%)	0.59 [0.56;0.63]	<0.001
Pulmonary	4189 (9.68%)	864 (7.38%)	0.43 [0.40;0.47]	<0.001
Hospital:				
No	22060 (51.0%)	5358 (45.7%)	Ref.	Ref.
Yes	21208 (49.0%)	6355 (54.3%)	1.23 [1.18;1.29]	<0.001
ICU:				
No	40654 (94.0%)	9047 (77.2%)	Ref.	Ref.
Yes	2614 (6.04%)	2666 (22.8%)	4.58 [4.32;4.86]	<0.001
hospital_mortality:				
No	38544 (89.1%)	10593 (90.4%)	Ref.	Ref.
Yes	4724 (10.9%)	1120 (9.56%)	0.86 [0.81;0.92]	<0.001
total_mortality:				
No	38332 (88.6%)	10496 (89.6%)	Ref.	Ref.
Yes	4936 (11.4%)	1217 (10.4%)	0.90 [0.84;0.96]	0.002
alert	13.6 (27.7)	12.2 (7.29)	1.00 [1.00;1.00]	<0.001
support	22.5 (18.7)	29.3 (10.3)	1.02 [1.02;1.02]	<0.001

	NEMSIS<br>N=43268	Sacyl<br>N=11713	Odds ratio [95%CI]	pvalue
transfer	18.4 (20.9)	12.5 (8.84)	0.97 [0.97;0.97]	<0.001
total	54.4 (48.4)	54.0 (17.1)	1.00 [1.00;1.00]	0.374
year:				
2018	2887 (6.67%)	1008 (8.61%)	Ref.	Ref.
2019	3946 (9.12%)	2073 (17.7%)	1.50 [1.38;1.65]	<0.001
2020	6594 (15.2%)	1305 (11.1%)	0.57 [0.52;0.62]	<0.001
2021	7264 (16.8%)	3330 (28.4%)	1.31 [1.21;1.43]	<0.001
2022	9027 (20.9%)	2858 (24.4%)	0.91 [0.83;0.99]	0.021
2023	13550 (31.3%)	1139 (9.72%)	0.24 [0.22;0.26]	<0.001
group:				
NEMSIS Rural	7069 (16.3%)	0 (0.00%)	Ref.	Ref.
NEMSIS Urban	36199 (83.7%)	0 (0.00%)	. [.;.]	.
Sacyl Rural	0 (0.00%)	2314 (19.8%)	. [.;.]	.
Sacyl Urban	0 (0.00%)	9399 (80.2%)	. [.;.]	.

Supplementary table S4. Comparison between the groups resulting from the combination of cohorts and rural and urban

	**NEMSIS Rural** N=7069	**NEMSIS Urban** N=36199	**Sacyl Rural** N=2314	**Sacyl Urban** N=9399	**pvalue**
age	61.7 (19.7)	60.4 (19.6)	62.8 (19.5)	65.4 (19.1)	<0.001
Sex:					0.001
Female	3009 (42.6%)	15214 (42.0%)	880 (38.0%)	3996 (42.5%)	
Male	4060 (57.4%)	20985 (58.0%)	1434 (62.0%)	5403 (57.5%)	
Level of Care of The EMS Unit:					<0.001
Basic life support	1746 (24.7%)	3184 (8.80%)	680 (29.4%)	3691 (39.3%)	
Advanced life support	5323 (75.3%)	33015 (91.2%)	1634 (70.6%)	5708 (60.7%)	
Zone:					<0.001
Rural	7069 (100%)	0 (0.00%)	2314 (100%)	0 (0.00%)	
Urban	0 (0.00%)	36199 (100%)	0 (0.00%)	9399 (100%)	
Complaint_Reported_by_Dispatch:					<0.001
causal_accident	1256 (17.8%)	5642 (15.6%)	118 (5.10%)	424 (4.51%)	
Disease	3067 (43.4%)	19469 (53.8%)	1757 (75.9%)	7717 (82.1%)	
Laboral_accident	3 (0.04%)	9 (0.02%)	59 (2.55%)	129 (1.37%)	
Others	1586 (22.4%)	4183 (11.6%)	21 (0.91%)	66 (0.70%)	
Social_demand	759 (10.7%)	4925 (13.6%)	137 (5.92%)	660 (7.02%)	
Traffic	398 (5.63%)	1971 (5.44%)	222 (9.59%)	403 (4.29%)	
Chief Complaint Organ System:					<0.001
Cardiovascular	1605 (22.7%)	8063 (22.3%)	879 (38.0%)	3761 (40.0%)	
Endocrine_Metabolic	164 (2.32%)	607 (1.68%)	45 (1.94%)	166 (1.77%)	
Gastrointestinal	240 (3.40%)	766 (2.12%)	98 (4.24%)	423 (4.50%)	
Genitourinary	88 (1.24%)	253 (0.70%)	4 (0.17%)	28 (0.30%)	
Global_General	1903 (26.9%)	13422 (37.1%)	175 (7.56%)	760 (8.09%)	
Lymphatic/Immune	30 (0.42%)	199 (0.55%)	135 (5.83%)	624 (6.64%)	
Musculoskeletal/Skin/Trauma	982 (13.9%)	2887 (7.98%)	433 (18.7%)	1080 (11.5%)	
Neurologic	1179 (16.7%)	6691 (18.5%)	416 (18.0%)	1822 (19.4%)	
Pulmonary	878 (12.4%)	3311 (9.15%)	129 (5.57%)	735 (7.82%)	
Hospital:					<0.001
No	6008 (85.0%)	16052 (44.3%)	935 (40.4%)	4423 (47.1%)	

	NEMSIS Rural N=7069	NEMSIS Urban N=36199	Sacyl Rural N=2314	Sacyl Urban N=9399	pvalue
Yes	1061 (15.0%)	20147 (55.7%)	1379 (59.6%)	4976 (52.9%)	
ICU:					<0.001
No	6453 (91.3%)	34201 (94.5%)	1620 (70.0%)	7427 (79.0%)	
Yes	616 (8.71%)	1998 (5.52%)	694 (30.0%)	1972 (21.0%)	
hospital_mortality:					<0.001
No	6541 (92.5%)	32003 (88.4%)	2071 (89.5%)	8522 (90.7%)	
Yes	528 (7.47%)	4196 (11.6%)	243 (10.5%)	877 (9.33%)	
total_mortality:					<0.001
No	6532 (92.4%)	31800 (87.8%)	2056 (88.9%)	8440 (89.8%)	
Yes	537 (7.60%)	4399 (12.2%)	258 (11.1%)	959 (10.2%)	
alert	16.5 (23.4)	13.1 (28.4)	22.8 (8.68)	9.61 (3.64)	<0.001
support	22.8 (22.7)	22.4 (17.8)	28.7 (11.1)	29.5 (10.1)	<0.001
transfer	25.8 (30.9)	16.9 (18.0)	21.1 (11.1)	10.4 (6.65)	<0.001
total	65.0 (55.1)	52.4 (46.7)	72.7 (19.5)	49.5 (12.9)	<0.001
year:					<0.001
2018	1090 (15.4%)	1797 (4.96%)	108 (4.67%)	900 (9.58%)	
2019	1167 (16.5%)	2779 (7.68%)	225 (9.72%)	1848 (19.7%)	
2020	1182 (16.7%)	5412 (15.0%)	308 (13.3%)	997 (10.6%)	
2021	1046 (14.8%)	6218 (17.2%)	838 (36.2%)	2492 (26.5%)	
2022	1307 (18.5%)	7720 (21.3%)	581 (25.1%)	2277 (24.2%)	
2023	1277 (18.1%)	12273 (33.9%)	254 (11.0%)	885 (9.42%)	
cohort:					<0.001
NEMSIS	7069 (100%)	36199 (100%)	0 (0.00%)	0 (0.00%)	
Sacyl	0 (0.00%)	0 (0.00%)	2314 (100%)	9399 (100%)	

Supplementary table S5. Patients' characteristics according to mortality for both cohorts rural patients

	Survivors N=8588	Non-survivors N=795	Odds ratio [95%CI]	pvalue
age	61.6 (19.7)	66.5 (18.7)	1.01 [1.01;1.02]	<0.001
Sex:				
Female	3597 (41.9%)	292 (36.7%)	Ref.	Ref.
Male	4991 (58.1%)	503 (63.3%)	1.24 [1.07;1.44]	0.005
Level of Care of This Unit:				
No	2253 (26.2%)	173 (21.8%)	Ref.	Ref.
Yes	6335 (73.8%)	622 (78.2%)	1.28 [1.07;1.53]	0.005
Zone: Rural	8588 (100%)	795 (100%)	Ref.	Ref.
Complaint_Reported_by_Dispatch:				
causal_accident	1272 (14.8%)	102 (12.8%)	Ref.	Ref.
Disease	4332 (50.4%)	492 (61.9%)	1.41 [1.14;1.78]	0.002
Laboral_accident	58 (0.68%)	4 (0.50%)	0.89 [0.26;2.23]	0.825
Others	1496 (17.4%)	111 (14.0%)	0.93 [0.70;1.22]	0.586
Social_demand	863 (10.0%)	33 (4.15%)	0.48 [0.32;0.71]	<0.001
Traffic	567 (6.60%)	53 (6.67%)	1.17 [0.82;1.64]	0.386
Chief Complaint Organ System:				
Cardiovascular	2155 (25.1%)	329 (41.4%)	Ref.	Ref.
Endocrine_Metabolic	205 (2.39%)	4 (0.50%)	. [.;.]	.
Gastrointestinal	316 (3.68%)	22 (2.77%)	. [.;.]	.
Genitourinary	92 (1.07%)	0 (0.00%)	. [.;.]	.
Global_General	1919 (22.3%)	159 (20.0%)	. [.;.]	.
Lymphatic/Immune	126 (1.47%)	39 (4.91%)	. [.;.]	.
Musculoskeletal/Skin/Trauma	1342 (15.6%)	73 (9.18%)	. [.;.]	.
Neurologic	1489 (17.3%)	106 (13.3%)	. [.;.]	.
Pulmonary	944 (11.0%)	63 (7.92%)	. [.;.]	.
Hospital:				

	Survivors N=8588	Non-survivors N=795	Odds ratio [95%CI]	pvalue
No	6492 (75.6%)	451 (56.7%)	Ref.	Ref.
Yes	2096 (24.4%)	344 (43.3%)	2.36 [2.04;2.74]	<0.001
ICU:				
No	7432 (86.5%)	641 (80.6%)	Ref.	Ref.
Yes	1156 (13.5%)	154 (19.4%)	1.55 [1.28;1.86]	<0.001
hospital_mortality:				
No	8588 (100%)	24 (3.02%)	Ref.	Ref.
Yes	0 (0.00%)	771 (97.0%)	. [.;.]	.
alert	18.1 (21.4)	16.9 (14.5)	1.00 [0.99;1.00]	0.091
support	24.2 (21.0)	24.7 (16.3)	1.00 [1.00;1.00]	0.470
transfer	25.1 (28.2)	18.9 (15.5)	0.99 [0.98;0.99]	<0.001
total	67.5 (50.0)	60.5 (34.7)	1.00 [0.99;1.00]	<0.001
year:				
2018	1087 (12.7%)	111 (14.0%)	Ref.	Ref.
2019	1296 (15.1%)	96 (12.1%)	0.73 [0.54;0.96]	0.027
2020	1345 (15.7%)	145 (18.2%)	1.06 [0.81;1.37]	0.684
2021	1735 (20.2%)	149 (18.7%)	0.84 [0.65;1.09]	0.188
2022	1736 (20.2%)	152 (19.1%)	0.86 [0.66;1.11]	0.241
2023	1389 (16.2%)	142 (17.9%)	1.00 [0.77;1.30]	0.995
cohort:				
NEMSIS	6532 (76.1%)	537 (67.5%)	Ref.	Ref.
Sacyl	2056 (23.9%)	258 (32.5%)	1.53 [1.30;1.78]	<0.001
group:				
NEMSIS Rural	6532 (76.1%)	537 (67.5%)	Ref.	Ref.
Sacyl Rural	2056 (23.9%)	258 (32.5%)	1.53 [1.30;1.78]	<0.001

Supplementary table S6. Patients' characteristics according to mortality for NEMSIS rural patients

	**Survivors** N=6532	**Non-survivors** N=537	**Odds ratio [95%CI]**	**pvalue**
age	61.7 (19.8)	62.6 (18.3)	1.00 [1.00;1.01]	0.303
Sex:				
Female	2822 (43.2%)	187 (34.8%)	Ref.	Ref.
Male	3710 (56.8%)	350 (65.2%)	1.42 [1.19;1.71]	<0.001
Level of Care of This Unit:				
No	1613 (24.7%)	133 (24.8%)	Ref.	Ref.
Yes	4919 (75.3%)	404 (75.2%)	1.00 [0.81;1.22]	0.964
Zone: Rural	6532 (100%)	537 (100%)	Ref.	Ref.
Complaint_Reported_by_Dispatch:				
causal_accident	1175 (18.0%)	81 (15.1%)	Ref.	Ref.
Disease	2776 (42.5%)	291 (54.2%)	. [.;.]	.
Laboral_accident	3 (0.05%)	0 (0.00%)	. [.;.]	.
Others	1480 (22.7%)	106 (19.7%)	. [.;.]	.
Social_demand	737 (11.3%)	22 (4.10%)	. [.;.]	.
Traffic	361 (5.53%)	37 (6.89%)	. [.;.]	.
Chief Complaint Organ System:				
Cardiovascular	1348 (20.6%)	257 (47.9%)	Ref.	Ref.
Endocrine_Metabolic	163 (2.50%)	1 (0.19%)	. [.;.]	.
Gastrointestinal	231 (3.54%)	9 (1.68%)	. [.;.]	.
Genitourinary	88 (1.35%)	0 (0.00%)	. [.;.]	.
Global_General	1746 (26.7%)	157 (29.2%)	. [.;.]	.
Lymphatic/Immune	29 (0.44%)	1 (0.19%)	. [.;.]	.
Musculoskeletal/Skin/Trauma	960 (14.7%)	22 (4.10%)	. [.;.]	.
Neurologic	1125 (17.2%)	54 (10.1%)	. [.;.]	.
Pulmonary	842 (12.9%)	36 (6.70%)	. [.;.]	.

	Survivors N=6532	Non-survivors N=537	Odds ratio [95%CI]	pvalue
Hospital:				
No	5564 (85.2%)	444 (82.7%)	Ref.	Ref.
Yes	968 (14.8%)	93 (17.3%)	1.21 [0.95;1.52]	0.124
ICU:				
No	5928 (90.8%)	525 (97.8%)	Ref.	Ref.
Yes	604 (9.25%)	12 (2.23%)	0.23 [0.12;0.39]	<0.001
hospital_mortality:				
No	6532 (100%)	9 (1.68%)	Ref.	Ref.
Yes	0 (0.00%)	528 (98.3%)	. [.;.]	.
alert	16.7 (23.9)	13.9 (15.9)	0.99 [0.99;1.00]	0.004
support	22.9 (23.1)	21.0 (16.8)	0.99 [0.99;1.00]	0.044
transfer	26.5 (31.7)	16.7 (16.5)	0.98 [0.98;0.99]	<0.001
total	66.1 (56.3)	51.6 (36.6)	0.99 [0.99;1.00]	<0.001
year:				
2018	988 (15.1%)	102 (19.0%)	Ref.	Ref.
2019	1101 (16.9%)	66 (12.3%)	0.58 [0.42;0.80]	0.001
2020	1087 (16.6%)	95 (17.7%)	0.85 [0.63;1.13]	0.265
2021	971 (14.9%)	75 (14.0%)	0.75 [0.55;1.02]	0.067
2022	1215 (18.6%)	92 (17.1%)	0.73 [0.55;0.98]	0.039
2023	1170 (17.9%)	107 (19.9%)	0.89 [0.67;1.18]	0.404
cohort: NEMSIS	6532 (100%)	537 (100%)	Ref.	Ref.

Supplementary table S7. Patients' characteristics according to mortality for Sacyl rural patients

	Survivors<br>N=2056	Non-survivors<br>N=258	Odds ratio [95%CI]	pvalue
age	61.3 (19.3)	74.7 (16.8)	1.05 [1.04;1.06]	<0.001
Sex:				
Female	775 (37.7%)	105 (40.7%)	Ref.	Ref.
Male	1281 (62.3%)	153 (59.3%)	0.88 [0.68;1.15]	0.350
Level of Care of This Unit:				
No	640 (31.1%)	40 (15.5%)	Ref.	Ref.
Yes	1416 (68.9%)	218 (84.5%)	2.45 [1.75;3.53]	<0.001
Zone: Rural	2056 (100%)	258 (100%)	Ref.	Ref.
Complaint_Reported_by_Dispatch:				
causal_accident	97 (4.72%)	21 (8.14%)	Ref.	Ref.
Disease	1556 (75.7%)	201 (77.9%)	0.59 [0.37;1.00]	0.049
Laboral_accident	55 (2.68%)	4 (1.55%)	0.35 [0.09;0.98]	0.045
Others	16 (0.78%)	5 (1.94%)	1.46 [0.43;4.28]	0.518
Social_demand	126 (6.13%)	11 (4.26%)	0.41 [0.18;0.87]	0.021
Traffic	206 (10.0%)	16 (6.20%)	0.36 [0.18;0.72]	0.004
Chief Complaint Organ System:				
Cardiovascular	807 (39.3%)	72 (27.9%)	Ref.	Ref.
Endocrine_Metabolic	42 (2.04%)	3 (1.16%)	. [.;.]	.
Gastrointestinal	85 (4.13%)	13 (5.04%)	. [.;.]	.
Genitourinary	4 (0.19%)	0 (0.00%)	. [.;.]	.
Global_General	173 (8.41%)	2 (0.78%)	. [.;.]	.
Lymphatic/Immune	97 (4.72%)	38 (14.7%)	. [.;.]	.
Musculoskeletal/Skin/Trauma	382 (18.6%)	51 (19.8%)	. [.;.]	.
Neurologic	364 (17.7%)	52 (20.2%)	. [.;.]	.
Pulmonary	102 (4.96%)	27 (10.5%)	. [.;.]	.
Hospital:				

	Survivors<br>N=2056	Non-survivors<br>N=258	Odds ratio [95%CI]	pvalue
No	928 (45.1%)	7 (2.71%)	Ref.	Ref.
Yes	1128 (54.9%)	251 (97.3%)	28.8 [14.6;68.2]	<0.001
ICU:				
No	1504 (73.2%)	116 (45.0%)	Ref.	Ref.
Yes	552 (26.8%)	142 (55.0%)	3.33 [2.56;4.35]	<0.001
hospital_mortality:				
No	2056 (100%)	15 (5.81%)	Ref.	Ref.
Yes	0 (0.00%)	243 (94.2%)	. [.;.]	.
alert	22.8 (8.74)	23.1 (8.23)	1.00 [0.99;1.02]	0.653
support	28.2 (10.9)	32.5 (12.2)	1.04 [1.02;1.05]	<0.001
transfer	20.8 (10.9)	23.5 (12.0)	1.02 [1.01;1.03]	<0.001
total	71.8 (19.2)	79.1 (20.4)	1.02 [1.01;1.02]	<0.001
year:				
2018	99 (4.82%)	9 (3.49%)	Ref.	Ref.
2019	195 (9.48%)	30 (11.6%)	1.67 [0.79;3.89]	0.187
2020	258 (12.5%)	50 (19.4%)	2.10 [1.04;4.75]	0.039
2021	764 (37.2%)	74 (28.7%)	1.05 [0.53;2.33]	0.894
2022	521 (25.3%)	60 (23.3%)	1.25 [0.63;2.79]	0.545
2023	219 (10.7%)	35 (13.6%)	1.73 [0.83;4.00]	0.147
cohort: Sacyl	2056 (100%)	258 (100%)	Ref.	Ref.

Supplementary table S8. Patients' characteristics according to mortality for both cohorts urban patients

	**Survivors** N=40240	**Non-survivors** N=5358	**Odds ratio [95%CI]**	**pvalue**
age	60.6 (19.7)	67.4 (17.6)	1.02 [1.02;1.02]	<0.001
Sex:				
Female	17139 (42.6%)	2071 (38.7%)	Ref.	Ref.
Male	23101 (57.4%)	3287 (61.3%)	1.18 [1.11;1.25]	<0.001
Level of Care of This Unit:				
No	6109 (15.2%)	766 (14.3%)	Ref.	Ref.
Yes	34131 (84.8%)	4592 (85.7%)	1.07 [0.99;1.16]	0.088
Zone: Urban	40240 (100%)	5358 (100%)	Ref.	Ref.
Complaint_Reported_by_Dispatch:				
causal_accident	5560 (13.8%)	506 (9.44%)	Ref.	Ref.
Disease	23271 (57.8%)	3915 (73.1%)	1.85 [1.68;2.04]	<0.001
Laboral_accident	128 (0.32%)	10 (0.19%)	0.87 [0.42;1.59]	0.673
Others	3805 (9.46%)	444 (8.29%)	1.28 [1.12;1.47]	<0.001
Social_demand	5316 (13.2%)	269 (5.02%)	0.56 [0.48;0.65]	<0.001
Traffic	2160 (5.37%)	214 (3.99%)	1.09 [0.92;1.29]	0.320
Chief Complaint Organ System:				
Cardiovascular	9374 (23.3%)	2450 (45.7%)	Ref.	Ref.
Endocrine_Metabolic	723 (1.80%)	50 (0.93%)	0.27 [0.20;0.35]	<0.001
Gastrointestinal	1116 (2.77%)	73 (1.36%)	0.25 [0.20;0.32]	<0.001
Genitourinary	277 (0.69%)	4 (0.07%)	0.06 [0.02;0.13]	<0.001
Global_General	12748 (31.7%)	1434 (26.8%)	0.43 [0.40;0.46]	<0.001
Lymphatic/Immune	681 (1.69%)	142 (2.65%)	0.80 [0.66;0.96]	0.016
Musculoskeletal/Skin/Trauma	3739 (9.29%)	228 (4.26%)	0.23 [0.20;0.27]	<0.001
Neurologic	7896 (19.6%)	617 (11.5%)	0.30 [0.27;0.33]	<0.001
Pulmonary	3686 (9.16%)	360 (6.72%)	0.37 [0.33;0.42]	<0.001
Hospital:				
No	18561 (46.1%)	1914 (35.7%)	Ref.	Ref.

	Survivors N=40240	Non-survivors N=5358	Odds ratio [95%CI]	pvalue
Yes	21679 (53.9%)	3444 (64.3%)	1.54 [1.45;1.63]	<0.001
ICU:				
No	36814 (91.5%)	4814 (89.8%)	Ref.	Ref.
Yes	3426 (8.51%)	544 (10.2%)	1.21 [1.10;1.33]	<0.001
hospital_mortality:				
No	40240 (100%)	285 (5.32%)	Ref.	Ref.
Yes	0 (0.00%)	5073 (94.7%)	. [.;.]	.
alert	12.8 (26.7)	9.24 (10.8)	0.98 [0.98;0.98]	<0.001
support	23.5 (16.8)	26.3 (16.0)	1.01 [1.01;1.01]	<0.001
transfer	16.0 (17.2)	12.4 (9.68)	0.98 [0.97;0.98]	<0.001
total	52.3 (43.8)	47.9 (25.3)	1.00 [1.00;1.00]	<0.001
year:				
2018	2383 (5.92%)	314 (5.86%)	Ref.	Ref.
2019	4216 (10.5%)	411 (7.67%)	0.74 [0.63;0.86]	<0.001
2020	5827 (14.5%)	582 (10.9%)	0.76 [0.66;0.88]	<0.001
2021	7876 (19.6%)	834 (15.6%)	0.80 [0.70;0.92]	0.002
2022	8698 (21.6%)	1299 (24.2%)	1.13 [0.99;1.29]	0.060
2023	11240 (27.9%)	1918 (35.8%)	1.29 [1.14;1.47]	<0.001
cohort:				
NEMSIS	31800 (79.0%)	4399 (82.1%)	Ref.	Ref.
Sacyl	8440 (21.0%)	959 (17.9%)	0.82 [0.76;0.88]	<0.001
group:				
NEMSIS Urban	31800 (79.0%)	4399 (82.1%)	Ref.	Ref.
Sacyl Urban	8440 (21.0%)	959 (17.9%)	0.82 [0.76;0.88]	<0.001

Supplementary table S9. Patients' characteristics according to mortality for NEMSIS urban patients

	**Survivors** N=31800	**Non-survivors** N=4399	**Odds ratio [95%CI]**	**pvalue**
age	59.6 (19.7)	65.8 (17.8)	1.02 [1.02;1.02]	<0.001
Sex:				
Female	13523 (42.5%)	1691 (38.4%)	Ref.	Ref.
Male	18277 (57.5%)	2708 (61.6%)	1.18 [1.11;1.26]	<0.001
Level of Care of This Unit:				
No	2610 (8.21%)	574 (13.0%)	Ref.	Ref.
Yes	29190 (91.8%)	3825 (87.0%)	0.60 [0.54;0.66]	<0.001
Zone: Urban	31800 (100%)	4399 (100%)	Ref.	Ref.
Complaint_Reported_by_Dispatch:				
causal_accident	5202 (16.4%)	440 (10.0%)	Ref.	Ref.
Disease	16368 (51.5%)	3101 (70.5%)	2.24 [2.02;2.49]	<0.001
Laboral_accident	7 (0.02%)	2 (0.05%)	3.56 [0.48;15.2]	0.180
Others	3749 (11.8%)	434 (9.87%)	1.37 [1.19;1.57]	<0.001
Social_demand	4699 (14.8%)	226 (5.14%)	0.57 [0.48;0.67]	<0.001
Traffic	1775 (5.58%)	196 (4.46%)	1.31 [1.09;1.56]	0.004
Chief Complaint Organ System:				
Cardiovascular	5916 (18.6%)	2147 (48.8%)	Ref.	Ref.
Endocrine_Metabolic	581 (1.83%)	26 (0.59%)	0.12 [0.08;0.18]	<0.001
Gastrointestinal	733 (2.31%)	33 (0.75%)	0.12 [0.09;0.17]	<0.001
Genitourinary	249 (0.78%)	4 (0.09%)	0.05 [0.01;0.11]	<0.001
Global_General	12009 (37.8%)	1413 (32.1%)	0.32 [0.30;0.35]	<0.001
Lymphatic/Immune	191 (0.60%)	8 (0.18%)	0.12 [0.05;0.22]	<0.001
Musculoskeletal/Skin/Trauma	2765 (8.69%)	122 (2.77%)	0.12 [0.10;0.15]	<0.001
Neurologic	6293 (19.8%)	398 (9.05%)	0.17 [0.16;0.19]	<0.001
Pulmonary	3063 (9.63%)	248 (5.64%)	0.22 [0.19;0.26]	<0.001
Hospital:				
No	14166 (44.5%)	1886 (42.9%)	Ref.	Ref.

	Survivors N=31800	Non-survivors N=4399	Odds ratio [95%CI]	pvalue
Yes	17634 (55.5%)	2513 (57.1%)	1.07 [1.00;1.14]	0.036
ICU:				
No	29875 (93.9%)	4326 (98.3%)	Ref.	Ref.
Yes	1925 (6.05%)	73 (1.66%)	0.26 [0.21;0.33]	<0.001
hospital_mortality:				
No	31800 (100%)	203 (4.61%)	Ref.	Ref.
Yes	0 (0.00%)	4196 (95.4%)	. [.;.]	.
alert	13.6 (29.9)	9.16 (11.8)	0.98 [0.97;0.98]	<0.001
support	22.1 (17.9)	24.7 (16.4)	1.01 [1.01;1.01]	<0.001
transfer	17.5 (18.7)	12.6 (10.1)	0.97 [0.97;0.97]	<0.001
total	53.2 (48.8)	46.5 (26.9)	1.00 [0.99;1.00]	<0.001
year:				
2018	1592 (5.01%)	205 (4.66%)	Ref.	Ref.
2019	2549 (8.02%)	230 (5.23%)	0.70 [0.57;0.85]	<0.001
2020	4950 (15.6%)	462 (10.5%)	0.72 [0.61;0.86]	<0.001
2021	5611 (17.6%)	607 (13.8%)	0.84 [0.71;1.00]	0.044
2022	6632 (20.9%)	1088 (24.7%)	1.27 [1.09;1.50]	0.002
2023	10466 (32.9%)	1807 (41.1%)	1.34 [1.15;1.57]	<0.001
cohort: NEMSIS	31800 (100%)	4399 (100%)	Ref.	Ref.

Supplementary table S10. Patients' characteristics according to mortality for Sacyl urban patients

	Survivors N=8440	Non-survivors N=959	Odds ratio [95%CI]	pvalue
age	64.3 (19.3)	74.8 (15.0)	1.04 [1.03;1.04]	<0.001
Sex:				
Female	3616 (42.8%)	380 (39.6%)	Ref.	Ref.
Male	4824 (57.2%)	579 (60.4%)	1.14 [1.00;1.31]	0.056
Level of Care of This Unit:				
No	3499 (41.5%)	192 (20.0%)	Ref.	Ref.
Yes	4941 (58.5%)	767 (80.0%)	2.83 [2.40;3.34]	<0.001
Zone: Urban	8440 (100%)	959 (100%)	Ref.	Ref.
Complaint_Reported_by_Dispatch:				
causal_accident	358 (4.24%)	66 (6.88%)	Ref.	Ref.
Disease	6903 (81.8%)	814 (84.9%)	0.64 [0.49;0.85]	0.002
Laboral_accident	121 (1.43%)	8 (0.83%)	0.37 [0.16;0.74]	0.004
Others	56 (0.66%)	10 (1.04%)	0.98 [0.45;1.95]	0.956
Social_demand	617 (7.31%)	43 (4.48%)	0.38 [0.25;0.57]	<0.001
Traffic	385 (4.56%)	18 (1.88%)	0.26 [0.14;0.43]	<0.001
Chief Complaint Organ System:				
Cardiovascular	3458 (41.0%)	303 (31.6%)	Ref.	Ref.
Endocrine_Metabolic	142 (1.68%)	24 (2.50%)	. [.;.]	.
Gastrointestinal	383 (4.54%)	40 (4.17%)	. [.;.]	.
Genitourinary	28 (0.33%)	0 (0.00%)	. [.;.]	.
Global_General	739 (8.76%)	21 (2.19%)	. [.;.]	.
Lymphatic/Immune	490 (5.81%)	134 (14.0%)	. [.;.]	.
Musculoskeletal/Skin/Trauma	974 (11.5%)	106 (11.1%)	. [.;.]	.
Neurologic	1603 (19.0%)	219 (22.8%)	. [.;.]	.
Pulmonary	623 (7.38%)	112 (11.7%)	. [.;.]	.
Hospital:				
No	4395 (52.1%)	28 (2.92%)	Ref.	Ref.
Yes	4045 (47.9%)	931 (97.1%)	35.9 [25.1;53.7]	<0.001

	Survivors N=8440	Non-survivors N=959	Odds ratio [95%CI]	pvalue
ICU:				
No	6939 (82.2%)	488 (50.9%)	Ref.	Ref.
Yes	1501 (17.8%)	471 (49.1%)	4.46 [3.88;5.12]	<0.001
hospital_mortality:				
No	8440 (100%)	82 (8.55%)	Ref.	Ref.
Yes	0 (0.00%)	877 (91.4%)	. [.;.]	.
alert	9.61 (3.63)	9.62 (3.73)	1.00 [0.98;1.02]	0.978
support	29.1 (9.83)	33.2 (11.8)	1.04 [1.03;1.05]	<0.001
transfer	10.3 (6.57)	11.3 (7.29)	1.02 [1.01;1.03]	<0.001
total	48.9 (12.6)	54.1 (14.5)	1.03 [1.02;1.03]	<0.001
year:				
2018	791 (9.37%)	109 (11.4%)	Ref.	Ref.
2019	1667 (19.8%)	181 (18.9%)	0.79 [0.61;1.02]	0.066
2020	877 (10.4%)	120 (12.5%)	0.99 [0.75;1.31]	0.959
2021	2265 (26.8%)	227 (23.7%)	0.73 [0.57;0.93]	0.011
2022	2066 (24.5%)	211 (22.0%)	0.74 [0.58;0.95]	0.018
2023	774 (9.17%)	111 (11.6%)	1.04 [0.78;1.38]	0.782
cohort: Sacyl	8440 (100%)	959 (100%)	Ref.	Ref.

References

1. Vandenbroucke JP. von Elm E. Altman DG. Gøtzsche PC. Mulrow CD. Pocock SJ. Poole C. Schlesselman JJ. Egger M; STROBE Initiative. Strengthening the Reporting of Observational Studies in Epidemiology (STROBE): explanation and elaboration. PLoS Med. 2007 Oct 16;4(10):e297.
